# Disturbance‐modulated symbioses in termitophily

**DOI:** 10.1002/ece3.3601

**Published:** 2017-11-09

**Authors:** Ivan Monteiro, Arleu Barbosa Viana‐Junior, Ricardo Ribeiro de Castro Solar, Frederico de Siqueira Neves, Og DeSouza

**Affiliations:** ^1^ Departamento de Ecologia Instituto de Ciências Biológicas Universidade Federal de Minas Gerais Belo Horizonte MG Brazil; ^2^ Pós‐graduação em Ecologia Departamento de Biologia Geral Universidade Federal de Viçosa Viçosa MG Brazil; ^3^ Departamento de Entomologia Universidade Federal de Viçosa Viçosa MG Brazil

**Keywords:** associational defense, fire, Habitat amelioration, inquilines, Isoptera, termites, termitophiles

## Abstract

Symbiosis, the living‐together of unlike organisms, underlies every major transition in evolution and pervades most ecological dynamics. Among examples of symbioses, the simultaneous occupation of a termite nest by its builder termites and intruding invertebrate species (so‐called termitophily) provides suitable macroscopic scenarios for the study of species coexistence in confined environments. Current evidence on termitophily abounds for dynamics occurring at the interindividual level within the termitarium, but is insufficient for broader scales such as the community and the landscape. Here, we inspect the effects of abiotic disturbance on termitophile presence and function in termitaria at these broader scales. To do so, we censused the termitophile communities inhabiting 30 termitaria of distinct volumes which had been exposed to increasing degrees of fire‐induced disturbance in a savanna‐like ecosystem in southeastern Brazil. We provide evidence that such an abiotic disturbance can ease the living‐together of termitophiles and termites. Putative processes facilitating these symbioses, however, varied according to the invader. For nonsocial invaders, disturbance seemed to boost coexistence with termites via the habitat amelioration that termitaria provided under wildfire, as suggested by the positive correlation between disturbance degree and termitophile abundance and richness. As for social invaders (ants), disturbance seemed to enhance associational defenses with termites, as suggested by the negative correlation between the presence of ant colonies and the richness and abundance of other termitarium‐cohabiting termitophiles. It is then apparent that disturbance‐modulated distinct symbioses in these termite nests.

## 1 INTRODUCTION

Symbiosis, understood as “the living‐together of unlike organisms” (De Bary, 1879), is a phenomenon underlying every major transition in evolution (Maynard‐Smith & Szathmáry, 1997) and pervading most ecological dynamics. It is not surprising, therefore, that examples of symbioses are spread throughout aquatic and terrestrial systems, at the microscopic and macroscopic scales. Among these latter, termitophily—the simultaneous occupation of termitaria by termites and other invertebrates—and its counterpart in ants, myrmecophily, provide macroscopic scenarios highly suitable for the study of species coexistence and community organization in confined environments. In spite of that, and differently from myrmecophily, the ecological processes underlying termitophily are still unclear. This is unfortunate, given the complementarity of termitophily in regard to myrmecophily: In the former, for instance, the nest is defended—if ever—by a detritivorous “landlord” while in the latter, such a role is played mostly by an omnivore. Processes and strategies underlying nest occupation can, hence, differ substantially between these two symbioses and their study should shed light on distinct ecological dynamics.

Current evidence on termitophily abounds for dynamics occurring at the interindividual level within the termitarium, but is insufficient for broader scales. Termitophily has been traditionally studied with a focus on one‐to‐one individual interactions, inspecting morphological and behavioral adaptations used by intruders to overcome barriers imposed by termite hosts (Grassé, 1986; Kistner, 1982). The respectable amount of knowledge thereby produced allowed, as a natural step forward, that the determinants of intruders’ success at the scale of the nest entered termitophily's research agenda (Cristaldo, Rosa, Florencio, Marins, & DeSouza, 2012; Leponce, Roisin, & Pasteels, 1999). We are now starting to perceive that intruders’ success in termitaria depends not only on interindividual interactions but also on factors operating at broader levels, viz., the nest or a set of nests (DeSouza et al., 2016; Marins et al., 2016). Adding this new focus to studies on termitophily is highly desirable, given the meaning of spatial scale on the colonization dynamics of patchy/insular environments, among which termitaria seem to fit quite well.

Following this trend, here we report on how disturbance at the level of the nest can affect species coexistence in termitaria. We consider that factors enhancing risks or reducing niche suitability in the surrounding environment should enhance the number of immigrants seeking shelter at a termitarium. In this regard, catastrophic events such as wildfires appear specially relevant to affect the rates at which intruders probe candidate nests because termitaria are effective refugia against fire (Avitabile, Nimmo, Bennett, & Clarke, 2015; DeSouza, Albuquerque, Tonello, Pinto, & Junior, 2003).

Arriving at a termite nest by no means guarantees establishment, as immigrants have to face local restrictions in the form of antagonistic interactions with the resident species, including termites (Marins et al., 2016) and other termitophiles such as ants (Higashi & Ito, 1989). Whereas mutual nest defense is not a rule among cohabiting termite species (Cristaldo, Rodrigues, Elliot, Araújo, & DeSouza, 2016), it has been reported quite convincingly for termitaria‐inhabiting ants (Higashi & Ito, 1989). Accordingly, in other symbiotic interactions, ants can act as biotic defenders; e.g., in ant–plant interactions, ants protect plants against herbivores and parasites (Janzen, 1966) and this effect is pronounced in nesting sites permanently inhabited by colonies of ants (Rosumek et al., 2009).

We hypothesize, hence, that symbiotic links within termitaria subjected to disturbance would be determined by the joint effects of these conflicting forces. By positively impacting immigration, disturbance would positively affect the number of termitophile individuals and, possibly, species in termitaria. This effect, however, should be counteracted by resident termites and ants, who would tend to impair newcomers’ establishment. Spatial constraints within nests would reinforce these interactions more stringently in smaller as opposed to larger termitaria. In short, we predict that the symbiotic linkage between termites and their cohabitants could be fine‐tuned by the ecological context to which their termitarium belongs.

## 2 MATERIAL AND METHODS

### 2.1 Ethics statement

All necessary permits were obtained for the described study, which complied with all relevant regulations of Brazil. This includes collecting and transportation permit from IBAMA (The Brazilian Institute for the Environment and Renewable Natural Resources), as well as tacit approval from the Brazilian Federal Government implied by granting the authors the post of Scientific Researcher.

### 2.2 Terms definition

We follow Momeni, Chen, Hillesland, Waite, and Shou (2011) in using De Bary's definition of symbiosis (i.e. “the living‐together of unlike organisms”), which encompasses any interaction along the full spectrum from reciprocal beneficial mutualism to antagonistic predator–prey interactions, via commensalism.

“Termites” are monophyletic social cockroaches (Blattodea) belonging to the Infra‐order Isoptera (Krishna, Grimaldi, Krishna, & Engel, 2013). The terms “termitarium” (plural: termitaria), “mound”, or “nest” denote the physical epigeic structure built by termites. “Colony” denotes the assemblage of individuals of a given social species (here, termites [Insecta: Blattodea] or ants [Insecta: Hymenoptera]) living and cooperating intraspecifically within a nest. Throughout this text, when we mention “termites” or “ants,” we are referring to a full colony of such insects. If at any point we need to refer to a given individual, we will explicitly say so.

“Coexistence” and “cohabitation” are used as synonyms and refer to the simultaneous occurrence of different species of invertebrates (including ants) within a given termitarium, without implication of reciprocal positive or negative influences. We generally refer to these as “termitophiles” but, for the sake of clarity, we will explicitly inform if we are referring to ants as needed. The term “intruder” may be used to refer to cohabitants simply because they establish themselves—not necessarily by force—in nests which had not been built by/for them in the first place. We may also, when referring to cohabitants, occasionally use the term “resident” to imply a cohabitant established in the nest, as opposed to “immigrant” or “newcomer, which will refer to organisms just arriving at the nest, regardless whether they belong to an established species or not. Termitaria‐inhabiting termites are normally referred to as “builders” or “inquilines” (Araujo, 1970), depending on whether they can be identified, respectively, as the original or secondary occupant of these nests. Here, the builder is very likely to be *Velocitermes heteropterus* (see subsection 2.4 and Section 2.1 below).

### 2.3 Overall experimental rationale

In order to inspect how would disturbance affect symbiotic links between termites and termitophiles, we censused the termitophile communities inhabiting 30 termitaria which had been exposed to increasing degrees of fire‐induced disturbance, 3 months before data collection.

These termitaria were located within a c.a. 1,000 ha valley, in a savanna‐like ecosystem in southeastern Brazil (GPS data presented on Table S1). At this location, winds blowing wildfires predominantly in one direction in seven distinct days created a gradient of heat and other effects across space, forming a well‐defined borderline between unburnt and burnt fields. This has inflicted distinct disturbance to any given termitarium according to its location within unburnt or burnt areas, allowing us to use the termitarium distance to the fire borderline as a proxy for the disturbance degree it was subjected to. After all, this line defines a hypothetical threshold defining the transition between milder and harsher disturbance degrees, being hence an useful reference point. In doing so, the extent of disturbance at the landscape level could be expressed as a continuous variable, ranging from negative to zero to positive values. The farther an unburnt termitarium was from the borderline (which as posed at zero), the milder the strain it was subjected to, and the more negative was the disturbance degree value assigned to this nest. Conversely, the deeper a termitarium was inside the burnt areas, the harsher the distress it was subjected to, and the more positive was the value assigned to it.

Simultaneously, at the local level, fire would also affect immigration/extinction dynamics in a termitarium because larger nests, being conspicuous, are easier targets for immigrants. Additionally, thicker walls of larger nests should provide better insulation and thence lower extinction rates.

Interactions between fire and nest volume are also bound to happen because immigration should be favored in larger as opposed to smaller nests because, in these latter, soldiers exert higher patrolling rates (DeSouza et al., 2016). Also, invaders are prone to be more successful in larger nests where space availability alleviates the rate and consequent harmful effects of interspecific encounters, particularly with termitophilous ants (Higashi & Ito, 1989).

In summary, disturbance inflicted by fire operates at two distinct scales which, combined, should determine the equilibrium number of species and individuals cohabiting a termite nest. At the regional scale, the distance to the fire event affects the number of immigrants seeking shelter at a termitarium, the closer to fire the larger should be these numbers. At the local level, the termitarium size would affect both, the immigrant numbers and the extinction rates of residents because, while larger nests are more conspicuous targets, they also ease coexistence by alleviating interspecific encounters between residents.

Simply put, the smaller the termitarium and the deeper it is within a burnt site, the higher the disturbance it should be subjected to. These two variables will compose our statistical model along with variables describing interspecific interactions.

Another important variable accountable for the termitophile loads is the time the termitarium has been exposed in the ecosystem, that is, the nest age. This is a very difficult parameter to estimate, being hence frequently omitted in ecological modeling. At least two variables can be accountable for nest age: the ontogentic stage of the termite colony and its population size (hence, nest volume), but neither of them alone or combined can provide a rigorous estimation of colony age. However, while not fully solving the problem of nest age estimation, keeping both variables as predictors of termitophile loads in termitaria would at least allow better partitioning of the deviance. Thinking of that, we have included in our statistical models, a covariate describing the presence or absence of termites alates in the studied termitaria. Because alates are only present in mature nests, this covariate would compete with the variable volume for the deviance due to nest age. Better details on the statistical analyses are given at the appropriate section below.

### 2.4 Focal species

*Velocitermes* spp. are exclusively neotropical termites, occurring between latitudes 20°N and 30°S, where they live in forests and savannas, foraging in the open at night and feeding on grass and leaf litter by means of trails departing from the nest over the surface of the ground. Such trails are subsequently transformed into covered runways after being continuously used (Coles De Negret & Redford, 1982; Mathews, 1977). *Velocitermes heteropterus* is a common species in Brazil, whose nests form a conical epigeous structure with soft and crumbly walls, built around a grass tussock (Coles De Negret & Redford, 1982, mistakenly referring to this species as *Velocitermes paucipilis* – D.E. Oliveira, personal communication). This species is known to live symbiotically with other termite species in the same termitarium (Florencio et al., 2013). When cohabiting with *Cornitermes snyderi*,* V. heteropterus* add tall earthen turrets on top of their host nest (Mathews, 1977).

### 2.5 Study site

The study was carried out in the Brazilian “cerrado”, an environment physiognomically but not floristically similar to a savanna, within a valley located at 800 m above sea level within the Serra do Cipó National Park (PARNA Cipó) which belongs to the Serra do Espinhaço Biosphere Reserve, in the municipality of Santana do Riacho, Minas Gerais State, southeastern Brazil (19°20°S; 43°44°W). In Köppen's classification, the study area is subjected to Cwb climate (subtropical highland climate) with well‐defined wet season (spring/summer) between October and April and dry season (autumn/winter) between May and September. Annual average temperature varies between 17 and 18.5°C, with annual rainfall between 1,450 and 1,800 mm.

Within this valley, burnt and unburnt termitaria were collected. While burnt termitaria had been subjected to wildfires between 06 and 12 October 2012, unburnt ones were in locales in which wildfires have not been recorded in the last 5 years. Predominant winds during the above period blown from the unburnt site toward southeast and the burnt field.

### 2.6 Data collection

A total of 30 termitaria have been collected in January 2013, 3 months after burning. Nests have been arbitrarily selected so that to choose those visually similar in size, color, and shape. No nest was selected within 50 m from another. All nests were epigeous with a shallow hypogeous portion. Before removing the whole nests (hypogeous + epigeous portions) from the field and taking them to the laboratory, their volumes were estimated by measuring the height and diameter of cylinders into which the mound was visually decomposed, as sketched by Cristaldo et al. (2012).

All invertebrates found in the nests were extracted by flotation in water and identified to the lowest taxonomic level as feasible. The extraction method followed Clarke and Garraway (1994) and consisted of two stages. At first, portions of the termite mounds were shaken by hand to dislodge the arthropods from the termite mound conducts. Then, these portions were further fragmented and immersed in saline water solution. The high density of the solution caused the organic matter, including termites and intruders present in termitarium, to float while the nest wall debris sunk, hence enabling easing separation of invertebrates from the precipitate.

### 2.7 Handling pseudoreplication

Pseudoreplication (sensu Hurlbert, 1984) so often and seriously compromises landscape‐level studies (Ramage et al., 2013) that we may disregard such studies prior to properly considering the statistical solutions eventually used (Davies & Gray, 2015; Oksanen, 2001). Here, we have chosen one of these solutions, avoiding the most typical flaw in fire‐induced disturbance studies, namely, to mistake interdependent readings for proper sampling units in two unreplicated treatments (burnt vs. unburnt). Rather, we follow Hurlbert's (2004, p. 594) advice using a treatment factor as a continuous variable (disturbance degree) and measuring the response variable (termitophile abundance or richness) on only one independent experimental unit (a termitarium) at each of the several levels of this treatment (the distance from the fire boundary coupled with the respective termitarium volume). We then fitted a regression model to such a dataset estimating error from the deviations of datapoints to the values predicted by the model, strictly as recommended by Hurlbert (2004). In doing so, we compared the 30 termitaria (and not sites) among each other in regard to the level of disturbance they've been exposed to, analyzing how would termitophile abundance and richness respond to such a disturbance.

### 2.8 Data analysis

To check for the effects of disturbance suffered by termitaria on their termitophile occupancy, we built two sets of candidate models, each set composed of models in which a single response variable (species richness or abundance of nonsocial termitophiles) was tested against the following explanatory variables: (1) the distance from termitarium to the borderline between burnt and unburnt areas, appearing in the models as dist and assuming negative values for termitaria left of the borderline and positive values otherwise; (2) the existence of a fully functional termitophilous ant colony in the termitarium, codified as ant and taking values 0 or 1 respectively for absence or presence; (3) the number of termite species inhabiting the termitarium, as tmt; (4) the external volume of the termitaria, or vol; and (5) the presence or absence of alate termites in the termitarium, codified as alt.

The candidate models have been based on the competing biological hypotheses (sensu Chamberlin, 1890) that the richness or abundance of termitophiles in termitaria would depend on:

1. a single factor, i.e., models containing a single explanatory variable; or
2. the simultaneous action of the factors, i.e., models containing a given set of the explanatory variables; or
3. the synergistic action of the factors, i.e., models containing a given set of the explanatory variables along with their statistical interactions. In this case, only second‐order interactions have been tested.

These models were submitted to “model selection,” a statistical approach differing from traditional null hypotheses testing in that it can be used to identify a single best model, thus lending support to one particular hypothesis, or it can be used to make inferences based on weighted support from a complete set of competing models. Analyses followed recommendations by Burnham and Anderson (2002) and were performed under R version 3.2.2 R Core Team (2015), using package MuMIn (Bartoń, 2016) under generalized linear modeling (GLM). Initially, a global model was built to encompass all candidate models for a given *y‐var* (richness or abundance of nonsocial termitophiles). Models have been built initially under Poisson errors and, when needed, corrected for overdispersion using Quasipoisson errors, being thence subjected to model selection. The fit and complexity of each candidate model were measured using second‐order Akaike information criterion (AIC_c_) modified to account for the dispersion parameter (QAIC_c_) when needed. Models were ranked to classify as the best the one presenting the lowest AIC_c_ or QAIC_c_. Inference on the existence of effects of explanatory variables on termitophiles’ parameters was made based on the subset of models whose AIC_c_ or QAIC_c_ differed from the best model's values by 2 or less units. Model averaging was applied to this subset of models, to build a final model including all variables whose averaged coefficients did not include zero within their standard error range. This final model was used to plot curves and derive the biological meaning explored in the Section 4.

## 3 RESULTS

We have recorded, in the 30 termitaria here studied, a total of 84 colonies from 15 termite (Insecta: Blattodea: Isoptera) (morpho)species and 19 colonies from two species of ants (Insecta: Hymenoptera: Formicidae), in addition to 958 individuals belonging to 100 morphospecies of nonsocial invertebrates from Arachnida, Collembola, Insecta, Malacostraca, and Myriapoda classes. The identities of these and their distribution among termitaria are presented on Table S2. The amount of individuals and the presence of eggs and juveniles in all ant colonies allowed the assumption that they were established as permanent lodgers in these termitaria.

All nests presented the same general architecture (conical and epigeous, see Section 2), closely resembling what is known for *V. heteropterus* nests. All nests showed no signs of senescence, such as damaged walls. *V. heteropterus* was the most abundant termite species found in 22 of these nests (Table S2). Among the remaining eight nests, five had *Cortaritermes rizzini* and three had *Silvestritermes euamignathus* as the most abundant termite species. All of these colonies were composed of lively workers and soldiers, some of them presenting alates. Species from these two latter genera normally build, respectively, characteristic low rounded or slightly dome‐shaped nests (Coles De Negret & Redford, 1982). It is then likely that the nests here studied have been all built by the same species (*V. heteropterus*) and secondarily occupied by others. That is to say, the currently abundant species in a given termitarium may or may not be the nest builder. Regardless, termites themselves did not show any detectable effect upon termitophile abundance nor did they affect termitophile species richness: The variable tmt (describing the number of resident termite colonies) and the variable alt (describing the presence of alate termites in these nests) were not included in any of the models with substantial empirical evidence of Table 1 and, consequently, were absent of the averaged model of Table 2.

**Table 1:** Models with substantial empirical evidence (∆ ≤ 2) predicting termitophile abundance or termitophile richness within termitaria in savanna‐like fields at “Serra do Cipó”, southeastern Brazil. Explanatory variables include (1) the distance (dist) from this termitarium to the borderline between burnt and unburnt areas; (2) the presence or absence of a functional ant colony (ant) in the termitarium; (3) the external volume of the termitarium (vol); and (4) the presence of termite alates (alt). Models comprise a subset of all candidate models used to describe all competing hypotheses, as described in Section 2. Models are based on 30 independent observations and refer to a multiple regression with generalized linear models under Quasipoisson (abundance) or Poisson errors (richness) and log‐link function. adj*R* ^2^ = adjusted *R* ^2^, *df* = degrees of freedom used by the model, Loglik = log‐likelihood, (Q)AIC_c_ = second‐order Akaike information criterion (QAIC_c_ for the case of termitophile abundance and AIC_c_ for richness), ∆ = AIC_c_ difference between the model under concern and the best model, Weight = Akaike weight, that is, the likelihood of the present model being the best in the candidate set. Global model: y ~ ( ant + dst + vol + tmt )^2^ + alt, where tmt stands for the number of termite species in the termitarium and superscript 2 represents all second‐order interactions between these terms. The variable tmt was not present in any of the competing best models

Model	adj*R* ^2^	*df*	Loglik	(Q)AIC_c_	∆	Weight
*y* = termitophile abundance (error = quasipoisson)						
ant + dist + vol + ant:vol	1	5	−96.138	111.1	0.00	0.482
ant + dist + vol + ant:vol + alt	1	6	−93.479	111.9	0.80	0.323
ant + dist	1	3	−104.056	112.9	1.81	0.195
*y* = termitophile richness (error = poisson)						
ant	0.9931	2	−79.891	164.2	0.00	0.267
ant + vol + ant:vol	0.9941	4	−77.627	164.9	0.63	0.195
ant + dist	0.9935	3	−79.204	165.3	1.11	0.154
ant + dist + vol + ant:vol	0.9945	5	−76.508	165.5	1.29	0.140
and + alt	0.9934	3	−79.307	165.5	1.31	0.139
ant + dist + vol + ant:vol + dist:vol	0.9950	6	−75.216	166.1	1.86	0.105

**Table 2:** Conditional average for the models predicting termitophile abundance or termitophile richness within termitaria in savanna‐like fields at “Serra do Cipó”, southeastern Brazil. Only models presenting ∆AIC_c_ ≤ 2 in model selection procedure (Table 1) have been included. The minimal adequate model used to plot curves in Figure 1 was built using only the terms below presenting Pr(>|*z*|) < .05. Nonsignificant simple terms have been kept if they took part in significant interactions. Plotting models were, hence, y ~ ant + dist + vol + ant:vol for termitophile abundance and y ~ ant + vol + ant:vol for termitophile richness. In order to plot such models, coefficients have been re‐estimated keeping only the significant terms in the model. Codes for variables are the same as in Table 1

	Estimate	*SE*	Adjusted *SE*	*z* Value	Pr(>|*z*|)
*y* = termitophile abundance (error = quasipoisson)					
(Intercept)	4.29e+00	1.51e−01	1.56e−01	27.47	<2e−16***
antspresent	−2.09e+00	3.55e−01	3.63e−01	5.75	<2e−06***
distfire	1.55e−04	4.74e−05	4.98e−05	3.12	.0018**
vol	−6.22e−03	3.37e−03	3.54e−03	1.76	.0789
antspresent:vol	1.66e−02	5.37e−03	5.64e−03	2.94	.0033**
alatespresent	−1.90e−01	1.06e−01	1.11e−01	1.70	.0888
*y* = termitophile richness (error = poisson)					
(Intercept)	3.17e+00	1.40e−01	1.45e−01	21.84	<2e−16***
antspresent	−1.58e+00	3.60e−01	3.66e−01	4.31	2e−05***
vol	−4.26e−03	4.56e−03	4.76e−03	0.89	.371
antspresent:vol	1.36e−02	6.16e−03	6.46e−03	2.11	.035*
distfire	1.45e−04	1.58e−04	1.61e−04	0.90	.367
alatespresent	1.34e−01	1.23e−01	1.29e−01	1.04	.297
distfire:vol	−6.69e−06	4.27e−06	4.50e−06	1.49	.137

*P < 0.05, **P < 0.01, ***P < 0.001.

### 3.1 Termitophile abundance

**Figure 1:**
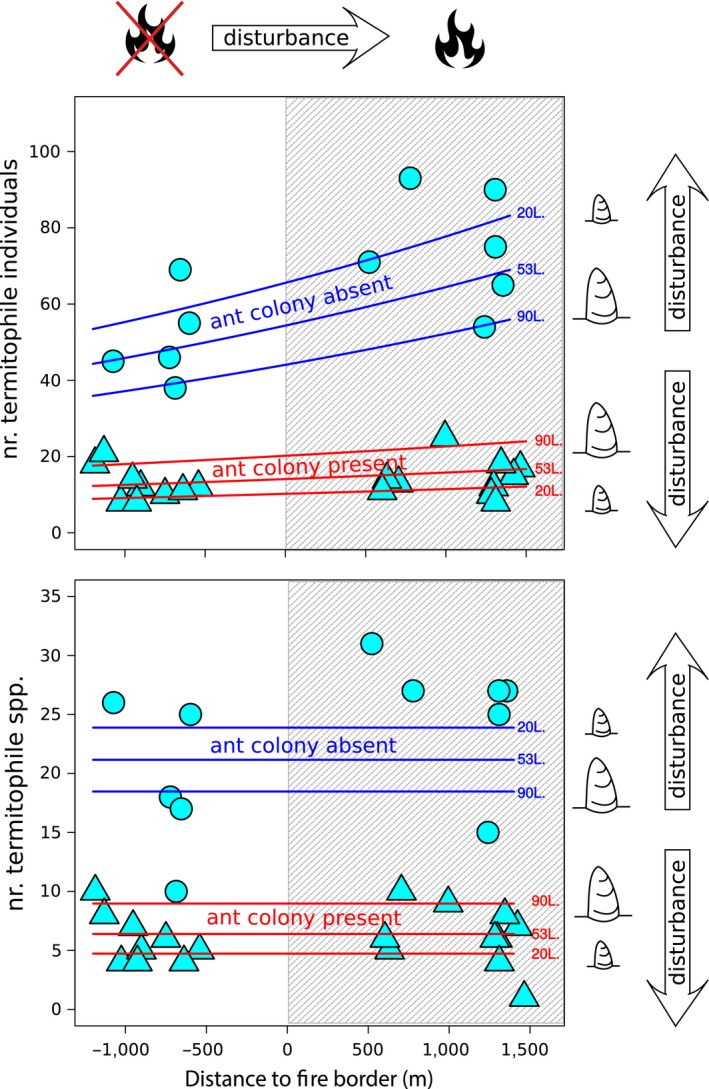
Combined effects the distance of termitaria to wildfire border, termitarium volume, and presence of ants on the abundance (top panel) and species richness (lower panel) of termitophiles cohabiting termitaria in savanna‐like fields in Serra do Cipó, southeastern Brazil. Hatched area evidences burnt termitaria. Circles represent termitaria housing no ant colony, and triangles are termitaria in which an ant colony was found. The *x*‐axis denotes increment in disturbance due to fire at the regional scale: Left side and negative values represent lower disturbance; right side and positive values represent higher disturbance. Disturbance also increases as volumes get smaller. See M&M for details

As predicted by our hypothesis, increments in degrees of disturbance positively affected the living‐together of termites and their termitophiles (Tables 1 and 2, Figure 1).

Termite mounds less affected by fire, held less nonsocial termitophile individuals than those mounds subjected to progressively harsher fire effects. Positive effects were also observed for local‐level disturbance: Smaller termitaria held more termitophile individuals than larger ones (Figure 1, upper panel, round plotting symbols). The absence of a statistical interaction between nest volume and distance to the fire borderline (Table 2) implies in independence between both variables.

Patterns of positive effects of disturbance on termitophile abundance in termitaria, however, have been strongly counteracted by the presence of an ant colony among such termitophiles. In such cases, the number of nonant termitophile individuals, although still rising with proximity of termitaria to fire, did so very slowly. More surprisingly, ants seemed able to revert completely the disturbance effects previously observed at the local scale. In termitaria bearing an ant colony (but not in antless ones), more termitophile individuals were observed in larger as opposed to smaller nests (Figure 1, upper panel, triangular plotting symbols). These results indicate that ants acted, in fact, as a barrier to the recruitment of other invertebrates to termitaria.

Such a “protective” role of resident ants in termitaria was more evident in burnt nests, as revealed by the increasing differences between “ant absent” and “ant present” curves in the upper panel of Figure 1. These differences occurred also in response to local‐level distress: The smaller the nest, the bigger the differences in the abundance of termitophiles between antless and ant‐bearing termitaria. Again, a positive correlation is apparent between disturbance degree and this alleged protective role of ants (Figure 1, upper panel).

In summary, termites coexisted with more termitophile individuals in termitaria which have been subjected to stronger disturbance, as long as no termitophilous ant colony was therein established. While symbiotic links between termites and termitophiles have been favored by landscape‐ and local‐scale stress, these same stresses strengthened the mitigating action of ants upon such links.

### 3.2 Termitophile species richness

Enhanced numbers of nonsocial termitophile individuals did not lead to enhanced species richness at the landscape level: For a given termitarium volume, the number of termitophile species was the same regardless the disturbance degree this termitarium has been subjected to (Figure 1, lower panel, horizontal lines).

Local‐level stress, however, positively affected termitophile richness: More species have been found in smaller, as opposed to larger, termitaria (Figure 1, lower panel, round plotting symbols). Again, ants were able to counteract positive effects of disturbance upon nonants. Far less nonsocial termitophile species have been found in termitaria bearing ant colonies than in antless ones. Among the former, the smaller their volume the lower the number of cohabitant species (Figure 1, lower panel, triangular plotting symbols). Distinct disturbance‐modulated symbioses can be seen in place again: Local distress favored termite—termitophile species cohabitation but under this same type of disturbance, termitophilous ants were able to impair nonsocial termitophile species establishment.

## 4 DISCUSSION

Our assessment of the rules imposed on termitophily by the ecological context surrounding the termitaria provides important hints to our understanding of the symbiotic links and the defense‐invading dynamics involving termites and termitophiles. Moreover, it extends to termites and their associated species, current empirical evidence on physical stress and disturbance as important drivers of symbioses (Seckbach & Grube, 2010) and species coexistence (Solar et al., 2015).

**Figure 2:**
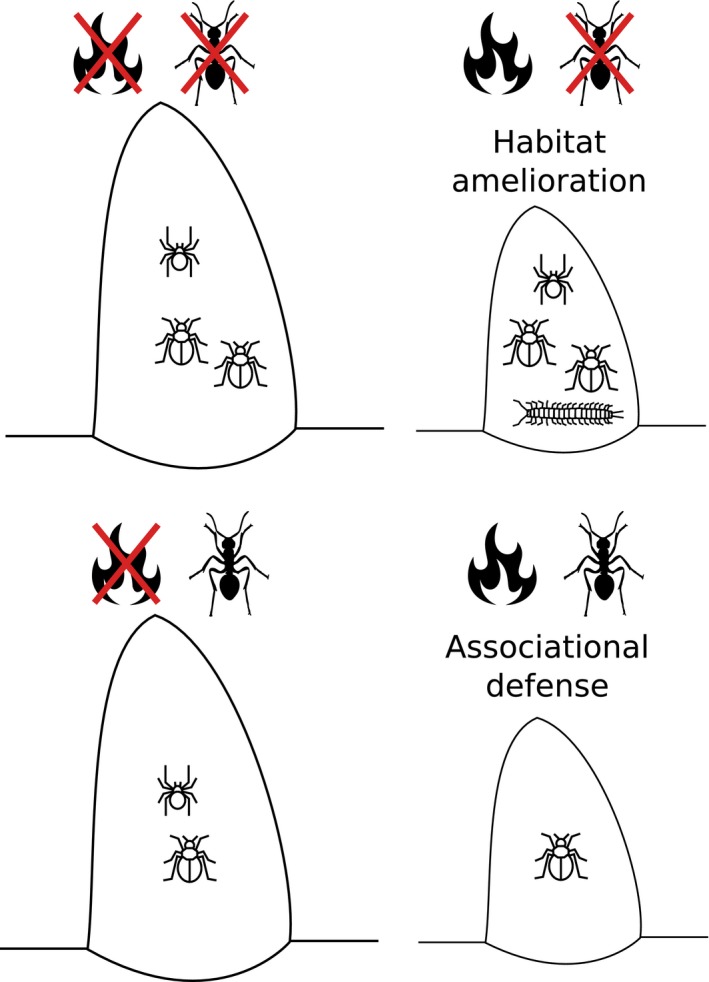
Pictorial summary of the main results. Under increasing disturbance (burning + small size), termitaria held larger termitophile abundance and richness, possibly due to habitat amelioration: Their insulating walls buffered fire or fire itself enhanced tolerance/lowered defense toward intruders on the part of termites. The presence of ants restrained termitophile recruitment and diversity, and this was boosted under stronger disturbance

Theoretically, stress is thought to strengthen symbiotic links through neighborhood habitat amelioration or associational defenses, and this is quite evident among sessile organisms such as barnacles, mussels, desert shrubs, terrestrial plants (Bertness & Callaway, 1994) and now, termite colonies (this paper). Both, habitat amelioration and associational defenses, are likely to be present in our system (Figures 1 and 2). Termitaria, with their insulating walls (Avitabile et al., 2015; DeSouza et al., 2003), should have provided an ameliorated habitat and the milder conditions may have pumped the invasion by other invertebrates at harsher locations, leading to the enhanced termitophile abundance and richness in these nests (Figure 1, Tables 1 and 2, see Section 2.1 for more details). This is in line with recent findings that abiotic factors do play important role on species establishment within (Marins et al., 2016) or upon termitaria (Traoré, Nygard, Guinko, & Lepage, 2008). Such an ameliorated habitat would also be formed if disturbance had enhanced tolerance or weakened defensive powers of the builder termite colony. This would seem plausible as stress, at least in the form of resource deprivation, attenuates intercolonial aggressivity and enhances neighbor tolerance in termites (Cristaldo, Araújo, et al., 2016; Cristaldo, Rodrigues, et al., 2016), easing colony fusion (Korb & Foster, 2010; Korb & Roux, 2012) and cooperative nest defense (Shellman Reeve, 1994).

Regardless whether enhanced cohabitation here observed had originated from increased tolerance or decreased defense on the part of termites, or from habitat amelioration alone, disturbance seems to have evidenced ants’ role as termitaria guarders. Ants living associated with termites seemed to be able to deter increasingly more termitophiles as termitaria were located progressively closer to harshness. It is tempting to consider these results as revealing a kind of “protection mutualism” (sensu Janzen, 1985) between ants and their host termites, such a mutualism being more and more intense with increasing disturbance. Such an idea finds support on earlier literature reporting that mutual defense of termitaria can be performed by cohabiting ants (Higashi & Ito, 1989) but not necessarily by cohabiting termite species (Cristaldo, Araújo, et al., 2016; Cristaldo, Rodrigues, et al., 2016). A similar protective role for predatory termitophiles has been already inferred by de Visser, Freymann, and Schnyder (2008) who have shown termitophile spiders (Arachnida) to not feed on termites directly, but on termitophagous invertebrates within the termitaria. Accordingly, effects of termitaria‐resident ants on their host termites have been shown to vary, from less to more negative, according to the ecological context (Holt & Greenslade, 1979).

Ants are well known to patrol and aggressively defend their colony territories and resources (Jongepier, Kleeberg, Job, & Foitzik, 2014), as demonstrated in studies with plants presenting some kind of pleasing resource to the ants, such as extrafloral nectaries. Such ants are effective enough to lower the number of herbivorous insects and hence the percentage of leaf damage, ultimately resulting in higher reproductive capacity of the patrolled plants (Rosumek et al., 2009; Trager et al., 2010). Among such ants, aggressiveness is escalated by the proximity of the menace to their nests (Oliveira, Oliveira Filho, & Cintra, 1987).

Specifically for the ants here studied, at least two unidentified congeneric species of *Camponotus rufipes* have been already described by Higashi and Ito (1989) to be highly dependent and eager defenders of their host nest, built by the termite *Armitermes laurensis*. *Camponotus rufipes* itself, a typical nectar‐feeding ant (Schilman & Roces, 2003), is very aggressive in defending resources, being highly efficient in locating and removing threats from their patrolled plants (Fagundes et al., 2017). Likewise, the seed harvester (Carroll & Risch, 1984) fire ant *Solenopsis geminata* also found in the termitaria here inspected, is notorious by its aggressive behavior (Wauters, Dekoninck, Herrera, & Fournier, 2014), preying upon vertebrates such as turtles (Wetterer & Lombard, 2010), birds hatchlings and chicks (Wetterer, 2011), and rats (Pimentel, 1955), being also able to reduce the diversity of arthropods in areas to which it has been introduced (Wetterer, 2011).

Being beyond the aim of this work, we have no direct observation of the mechanisms by which both *Camponotus rufipes* and *Solenopsis geminata* ants were able to deter termitophiles in the termitaria here studied. In spite of that, evidence provided above seems to support the notion that, when patrolling the area of their nests, such ants would also cover the surface of the host termitarium, chasing invaders and preventing them to settle in.

In the present case, affirming the existence of protection mutualism by such ants upon their termite hosts needs a necessary assumption that other termitophiles bring some burden to their termite hosts, but this remains speculative. In fact, one of the current weaknesses of studies on termitophily is the insufficient evidence to position termitophilic associations along the mutualism–parasitism continuum (but see, Campbell et al., 2016). That is the reason we employ the term “symbiosis” in its broadest sense, i.e., including negative, neutral and positive interactions (see Terms definition in Section 2). It is true that some termitophiles are known to predate upon termites but others may simply live as commensals (Grassé, 1986; Kistner, 1982). All in all, at least by taking over space originally built for termites’ nestmates, termitophiles can be viewed as intruders and, hence, their deterrence by termitophilous ants might be considered mutual nest defense.

In summary (Figure 2), it seems hence plausible to consider that disturbance would have played two important roles on termitophily in the nests here studied. On the one hand, it has eased cohabitation between termites and termitophiles thereby allowing higher cohabitant loads in termitaria. On the other, distress has attenuated eventual negative effects of termitophilous ants on their termite hosts (e.g., from predation), by offsetting losses with an associated benefit, namely enhanced refractoriness of termitaria to intruders. Thus, harshness affected cohabitation in termite nests providing distinct examples of “disturbance‐modulated symbioses”. The ecological context in general and distresses in particular should hence be incorporated in studies of interspecies associations under termitophily.

## AUTHOR CONTRIBUTIONS

IM involved in conception, design, acquisition of data, initial analyses, interpretation and drafting, revising final ms version, final approval. ABVJ involved in initial analyses and interpretation of data, initial drafting, revising final ms version, final approval. RRCS involved in analyses and interpretation of data, revising final ms version, final approval. FSN involved in conception, design, acquisition of data, initial analyses and interpretation, initial drafting, revising final ms version, final approval. ODS involved in conception, analyses and interpretation, drafting and revising latter versions, drawings, final approval.

## CONFLICT OF INTEREST

None declared.
