# Supplementary material for: Disturbance‐modulated symbioses in termitophily
Source: Ecol Evol. 2017 Nov 9;7(24):10829–38. doi: 10.1002/ece3.3601 (PMC5743531; doi:10.1002/ece3.3601)
Supplement: Supplementary file 1 [file ECE3-7-10829-s001.pdf]

Ivan Monteiro, Arleu Viana, Ricardo Ribeiro Castro Solar, Frederico Neves, Og DeSouza

Disturbance-modulated symbioses in termitophily

Corresponding author: Og DeSouza <og.souza@ufv.br>

Appendix Table 01: metadata for termitaria used in the above paper.

Each line is a single termitarium.

Codes in columns:

termitarium = the field code for the termitarium
utm_zone = UTM zone
utm_northing = UTM Norhing for the termitarium
utm_easting = UTM Easting for the termitarium
distance = distance from termitarium to the fire borderline (meters)
volume = termitarium's volume (litres)
alates = presence of alate termites in the termitarium

termitarium	utm_zone	utm_northing	utm_easting	distance	volume	alates
C01	23k	7857961.02	646842.7	777.5	35.1	absent
C02	23k	7857900.81	646801.5	705.1	33.2	present
C03	23k	7857870.65	646725.9	632.3	36.7	present
C04	23k	7857876.82	646675.3	603.2	32.6	absent
C05	23k	7857823.19	646613.7	522.2	24.1	absent
C06	23k	7859245.62	646433.9	1463.8	33.1	absent
C07	23k	7859231.04	646388.4	1422.6	48.3	present
C08	23k	7859176.35	646349.3	1355.8	33.4	present
C09	23k	7859139.63	646317.3	1307.1	29.3	absent
C10	23k	7859096.89	646264.8	1240.2	39.6	absent
C11	23k	7859859.03	645566.5	1344.0	29.5	absent
C12	23k	7859857.69	645498.8	1297.9	32.1	present
C13	23k	7859878.77	645451.7	1282.3	39.3	absent
C14	23k	7859952.14	645411.7	1310.4	31.0	present
C15	23k	7860001.64	645353.4	1308.5	35.0	absent
C16	23k	7853193.08	649524.7	994.5	77.2	absent
C17	23k	7853147.11	649529.3	-1025.7	39.1	absent
C18	23k	7853085.04	649527.0	-1073.6	77.2	absent
C19	23k	7852925.91	649535.6	-1186.6	93.2	absent
C20	23k	7852998.61	649537.7	-1130.9	44.1	absent
C21	23k	7853533.11	649283.5	-901.4	56.4	absent
C22	23k	7853467.51	649279.8	-952.8	55.8	absent
C23	23k	7853462.03	649326.0	-926.0	36.0	absent
C24	23k	7853845.49	649160.7	-750.0	69.6	present
C25	23k	7853896.65	649144.7	-722.6	19.1	absent
C26	23k	7854277.69	648989.1	-541.8	55.6	absent
C27	23k	7854236.06	648951.0	-598.3	19.4	present
C28	23k	7854210.52	648893.5	-655.6	22.9	absent
C29	23k	7854149.69	648915.0	-686.7	63.5	absent
C30	23k	7854152.77	648984.0	-638.4	19.7	absent
