# Supplementary material for: Disturbance‐modulated symbioses in termitophily
Source: Ecol Evol. 2017 Nov 9;7(24):10829–38. doi: 10.1002/ece3.3601 (PMC5743531; doi:10.1002/ece3.3601)
Supplement: Supplementary file 2 [file ECE3-7-10829-s002.pdf]

Appendix Table 2: Number of arthropod individuals cohabiting termitaria directly exposed to wildfire (C01 to C15) or located in neighbouring unburnt areas (c16 to c30) in a savanna-like ecosystem South-eastern Brazil (for ants and termites, the quantities refer to the number of colonies). See Appendix Table 1 for metadata on termitaria.

Class	Order	Family	(Morpho)species	C01	C02	C03	C04	C05	C06	C07	C08	C09	C10	C11	C12	C13	C14	C15	C16	C17	C18	C19	C20	C21	C22	C23	C24	C25	C26	C27	C28	C29	C30
Arachnida	Acarina	unident	Acari	8	1			3			5	4						3	2		3		1					8		12	5	11	
Arachnida	Araneae	Araneidae	Araneidae	1	1			1				1																					
Arachnida	Araneae	Corinnidae	Corinnidae_1		2			1					3																		1		
Arachnida	Araneae	Corinnidae	Corinnidae_2																		1												
Arachnida	Araneae	Ctenidae	Ctenidae_1	1				1				1									1												
Arachnida	Araneae	Ctenidae	Ctenidae_2	1				1																									
Arachnida	Araneae	Dipluridae	Dipluridae	1				1																									
Arachnida	Araneae	Gnaphosidae	Gnaphosidae																		1												
Arachnida	Araneae	Linyphiidae	Linyphiidae_1	1				1					1										3					2			3		
Arachnida	Araneae	Linyphiidae	Linyphiidae_2																									4			1		
Arachnida	Araneae	Lycosidae	Lycosidae					2																						1			
Arachnida	Araneae	Ochyroceratidae	Ochyroceratidae					2			1	2																			1		
Arachnida	Araneae	Oonopidae	Oonopidae_1	1				1										1															
Arachnida	Araneae	Oonopidae	Oonopidae_2								1							2					1					2			4		
Arachnida	Araneae	Palpimanidae	Palpimanidae_1								1																			1			
Arachnida	Araneae	Palpimanidae	Palpimanidae_2															1			1												
Arachnida	Araneae	Salticidae	Salticidae_1	1	1			3			3	3		1				2												1			
Arachnida	Araneae	Salticidae	Salticidae_2	3														1										3			1		
Arachnida	Araneae	Salticidae	Salticidae_3								1																						
Arachnida	Araneae	Tetrablemmidae	Tetrablemmidae									1																					
Arachnida	Araneae	Theridiidae	Theridiidae_1	1								1									1									1			
Arachnida	Araneae	Theridiidae	Theridiidae_2																											1			
Arachnida	Araneae	Zodariidae	Zodariidae								1							3			1									1			
Arachnida	Opiliones	Gonyleptidae	Gonyleptidae_1										1			2					1									1			
Arachnida	Opiliones	Gonyleptidae	Gonyleptidae_2				2						6										3		1			3			1	1	
Arachnida	Opiliones	unident	Opiliones_sp1	1				1				1										1											
Arachnida	Opiliones	unident	Opiliones_sp2																							1				1	1		
Arachnida	Opiliones	unident	Opiliones_sp3					1																									
Arachnida	Opiliones	Zalmoxidae	Zalmoxidae_1								4					1						1			1								
Arachnida	Opiliones	Zalmoxidae	Zalmoxidae_2	3								5						3			2	1											
Arachnida	Opiliones	Zalmoxidae	Zalmoxidae_3	1			1																							4			
Arachnida	Pseudoscorpiones	unident	Pseudoscorpiones	26	3			12		4	10	13	11			3		6			6									4			
Collembola	Entomobryomorpha	Entomobryidae	Entomobryidae_1	4																					1								
Collembola	Entomobryomorpha	Entomobryidae	Entomobryidae_2					3			1		14								2			3				7				7	
Collembola	Entomobryomorpha	Entomobryidae	Entomobryidae_3								1																			1			

Corresponding author: Og DeSouza <og.souza@ufv.>

Appendix Table 2: Number of arthropod individuals cohabiting termitaria directly exposed to wildfire (C01 to C15) or located in neighbouring unburnt areas (c16 to c30) in a savanna-like ecosystem South-eastern Brazil (for ants and termites, the quantities refer to the number of colonies). See Appendix Table 1 for metadata on termitaria.

Class	Order	Family	(Morpho)species	C01	C02	C03	C04	C05	C06	C07	C08	C09	C10	C11	C12	C13	C14	C15	C16	C17	C18	C19	C20	C21	C22	C23	C24	C25	C26	C27	C28	C29	C30
Collembola	Entomobryomorpha	Entomobryidae	Entomobryidae_4															3							2								
Collembola	Entomobryomorpha	Paronellidae	Paronellidae								1	18												2									
Collembola	Entomobryomorpha	Sminthurididae	Sminthurididae																												18		
Insecta	Blattodea	Blaberidae	Blaberidae								2																						
Insecta	Blattodea	Blatiidae	Blatiidae					1														2											
Insecta	Blattodea	Blattellidae	Blattellidae_1	1																							2						
Insecta	Blattodea	Blattellidae	Blattellidae_2	2				1			3	5		2				1			3	1								1			
Insecta	Blattodea	Blattellidae	Blattellidae_3	10				6			2	4						2			1									3			
Insecta	Blattodea	Blattellidae	Blattellidae_4							1	5				1												1					1	
Insecta	Blattodea_Isoptera	Rhinotermitidae	Heterotermes_sp								1																						
Insecta	Blattodea_Isoptera	Rhinotermitidae	Heterotermes tenuis			1											1																
Insecta	Blattodea_Isoptera	Termitidae	Cortaritermes_sp								1																						
Insecta	Blattodea_Isoptera	Termitidae	Cortaritermes rizinii	1		1					1			1	1	1	1		1	1	1		1	1			1	1			1		
Insecta	Blattodea_Isoptera	Termitidae	Dihoplotermes inusitatus	1										1	1	1	1			1							1	1					
Insecta	Blattodea_Isoptera	Termitidae	Diversitermes castaniceps		1																1												
Insecta	Blattodea_Isoptera	Termitidae	Embiratermes festivellus																													1	
Insecta	Blattodea_Isoptera	Termitidae	Labiotermes brevilabius								1																						
Insecta	Blattodea_Isoptera	Termitidae	Neocapritermes opacus														1																
Insecta	Blattodea_Isoptera	Termitidae	Parvitermes bacchanalis		1							1																					
Insecta	Blattodea_Isoptera	Termitidae	Silvestritermes euamignatus			1		1	1	1	1					1		1	1					1					1	1	1		
Insecta	Blattodea_Isoptera	Termitidae	Spinitermes_sp			1					1																					1	
Insecta	Blattodea_Isoptera	Termitidae	Subulitermes_sp											1								1										1	
Insecta	Blattodea_Isoptera	Termitidae	Termes ayri				1					1	1		1		1			1	1					1	1						1
Insecta	Blattodea_Isoptera	Termitidae	Velocitermes heteropterus	1	1	1	1	1		1	1	1	1			1		1	1		1	1	1	1	1	1	1	1			1	1	
Insecta	Coleoptera	Anthicidae	Anthicidae														1				1												
Insecta	Coleoptera	Carabidae	Carabidae								3	2						2										1					
Insecta	Coleoptera	Cleridae	Cleridae											2																1			
Insecta	Coleoptera	Curculionidae	Curculionidae_1									1																					
Insecta	Coleoptera	Curculionidae	Curculionidae_2													1																	
Insecta	Coleoptera	Curculionidae	Scolytinae_1												2								4	3				1	4		9		
Insecta	Coleoptera	Curculionidae	Scolytinae_2									2									2					4		3	1			5	
Insecta	Coleoptera	Derodontidae	Derodontidae					2								1					1												
Insecta	Coleoptera	Nitidulidae	Nitidulidae_1											3					2		1							1					
Insecta	Coleoptera	Nitidulidae	Nitidulidae_2										1																				
Insecta	Coleoptera	Nitidulidae	Nitidulidae_3																			3								1			

Corresponding author: Og DeSouza <og.souza@ufv.>

Appendix Table 2: Number of arthropod individuals cohabiting termitaria directly exposed to wildfire (C01 to C15) or located in neighbouring unburnt areas (c16 to c30) in a savanna-like ecosystem South-eastern Brazil (for ants and termites, the quantities refer to the number of colonies). See Appendix Table 1 for metadata on termitaria.

Class	Order	Family	(Morpho)species	C01	C02	C03	C04	C05	C06	C07	C08	C09	C10	C11	C12	C13	C14	C15	C16	C17	C18	C19	C20	C21	C22	C23	C24	C25	C26	C27	C28	C29	C30
Insecta	Coleoptera	Nitidulidae	Nitidulidae_4																											1	3		
Insecta	Coleoptera	Nitidulidae	Nitidulidae_5								1		1																				
Insecta	Coleoptera	Scarabaeidae	Scarabaeinae_1								1											2											
Insecta	Coleoptera	Scarabaeidae	Scarabaeinae_2		1																												
Insecta	Coleoptera	Staphylinidae	Staphylinidae_1								1									3	1						1						
Insecta	Coleoptera	Staphylinidae	Staphylinidae_2	2		5		1			1							1		3			1					3		2			
Insecta	Coleoptera	Staphylinidae	Staphylinidae_3										2																				
Insecta	Coleoptera	Staphylinidae	Staphylinidae_4								1																						
Insecta	Coleoptera	Staphylinidae	Staphylinidae_5		1										2										2			1			11	2	
Insecta	Coleoptera	Staphylinidae	Staphylinidae_6			4		2		4		4	1	3		2		23				4		3								2	
Insecta	Coleoptera	Staphylinidae	Staphylinidae_7									1			5															1			
Insecta	Coleoptera	Staphylinidae	Staphylinidae_8					1						1													2						
Insecta	Coleoptera	Tenebrionidae	Tenebrionidae_1		1								1												3								
Insecta	Coleoptera	Tenebrionidae	Tenebrionidae_2							1								2															
Insecta	Coleoptera	unident	Coleoptera_L1	1																													
Insecta	Coleoptera	unident	Coleoptera_L10																		1												
Insecta	Coleoptera	unident	Coleoptera_L11																													4	
Insecta	Coleoptera	unident	Coleoptera_L2							1									1														
Insecta	Coleoptera	unident	Coleoptera_L3				1			1								1			1												
Insecta	Coleoptera	unident	Coleoptera_L4																4	1													
Insecta	Coleoptera	unident	Coleoptera_L5																1														
Insecta	Coleoptera	unident	Coleoptera_L6									2																					
Insecta	Coleoptera	unident	Coleoptera_L7															3															
Insecta	Coleoptera	unident	Coleoptera_L8																		1												
Insecta	Coleoptera	unident	Coleoptera_L9																		2												
Insecta	Dermaptera	Labiidae	Labiidae			1							6														3	1			2		
Insecta	Diptera	unident	Diptera_L1				2				1						3	2	7								1						
Insecta	Embioptera	unident	Embioptera	1																													
Insecta	Hemiptera	Cydnidae	Cydnidae																									1				1	
Insecta	Hemiptera	unident	Auchenorrhyncha			3		6	17		3								6		3												4
Insecta	Hemiptera	unident	Hemiptera_1	1				1				2																	1				
Insecta	Hemiptera	unident	Hemiptera_L1										1																				
Insecta	Hemiptera	unident	Sternorrhyncha							3								3											4				
Insecta	Hymenoptera	Formicidae	Camponotus rufipes			1				1						1	1		1			1											
Insecta	Hymenoptera	Formicidae	Solenopsis geminata		1		1		1					1	1					1			1	1	1	1	1		1				1

Corresponding author: Og DeSouza <og.souza@ufv.>

Appendix Table 2: Number of arthropod individuals cohabiting termitaria directly exposed to wildfire (C01 to C15) or located in neighbouring unburnt areas (c16 to c30) in a savanna-like ecosystem South-eastern Brazil (for ants and termites, the quantities refer to the number of colonies). See Appendix Table 1 for metadata on termitaria.

Class	Order	Family	(Morpho)species	C01	C02	C03	C04	C05	C06	C07	C08	C09	C10	C11	C12	C13	C14	C15	C16	C17	C18	C19	C20	C21	C22	C23	C24	C25	C26	C27	C28	C29	C30
Insecta	Lepidoptera	unident	Lepdoptera_L1									1																					
Insecta	Orthoptera	unident	Orthoptera_L1			1															2									2			
Insecta	Orthoptera	unident	Orthoptera_L2												1											2				1			
Insecta	Psocoptera	unident	Psocoptera_1									2																					
Insecta	Psocoptera	unident	Psocoptera_2					1									2																
Insecta	Thysanoptera	unident	Thysanoptera				3	2				4						2												5			4
Malacostraca	Isopoda	Bathytropidae	Bathytropidae	1	1			3			2	3	3					1		1			3								1		
Malacostraca	Isopoda	Pudeoniscidae	Pudeoniscidae	6				1			4	2	2	2				4			2	1	5					2		2	1		2
Myriapoda	unident	unident	Myriapoda_1	11	1		2	5			5	4			1			2			3	2		1		1		2		5			1
Myriapoda	unident	unident	Myriapoda_2	2				3						4				1	1											1			
Myriapoda	unident	unident	Myriapoda_3					1				1					2								4			1			6	4	
Myriapoda	unident	unident	Myriapoda_4	1															1										2				

Corresponding author: Og DeSouza <og.souza@ufv.>
